# Multi-scale segmentation in GBM treatment using diffusion tensor imaging

**DOI:** 10.1016/j.compbiomed.2020.103815

**Published:** 2020-08

**Authors:** Roushanak Rahmat, Khadijeh Saednia, Mohammad Reza Haji Hosseini Khani, Mohamad Rahmati, Raj Jena, Stephen J. Price

**Affiliations:** a Department of Clinical Neuroscience, University of Cambridge, UK; b Department of Computer Engineering, Amirkabir University of Technology, Iran; c Department Electrical Engineering and Computer Science, York University, Canada; d Oncology Centre, Addenbrooke's Hospital, Cambridge, UK

**Keywords:** Image segmentation, Deep learning, GBM, DTI-MRI

## Abstract

Glioblastoma (GBM) is the commonest primary malignant brain tumor in adults, and despite advances in multi-modality therapy, the outlook for patients has changed little in the last 10 years. Local recurrence is the predominant pattern of treatment failure, hence improved local therapies (surgery and radiotherapy) are needed to improve patient outcomes. Currently segmentation of GBM for surgery or radiotherapy (RT) planning is labor intensive, especially for high-dimensional MR imaging methods that may provide more sensitive indicators of tumor phenotype. Automating processing and segmentation of these images will aid treatment planning. Diffusion tensor magnetic resonance imaging is a recently developed technique (DTI) that is exquisitely sensitive to the ordered diffusion of water in white matter tracts. Our group has shown that decomposition of the tensor information into the isotropic component (*p* – shown to represent tumor invasion) and the anisotropic component (*q* – shown to represent the tumor bulk) can provide valuable prognostic information regarding tumor infiltration and patient survival. However, tensor decomposition of DTI data is not commonly used for neurosurgery or radiotherapy treatment planning due to difficulties in segmenting the resultant image maps. For this reason, automated techniques for segmentation of tensor decomposition maps would have significant clinical utility. In this paper, we modified a well-established convolutional neural network architecture (CNN) for medical image segmentation and used it as an automatic multi-sequence GBM segmentation based on both DTI image maps (*p* and *q* maps) and conventional MRI sequences (T2-FLAIR and T1 weighted post contrast (T1c)). In this proof-of-concept work, we have used multiple MRI sequences, each with individually defined ground truths for better understanding of the contribution of each image sequence to the segmentation performance. The high accuracy and efficiency of our proposed model demonstrates the potential of utilizing diffusion MR images for target definition in precision radiation treatment planning and surgery in routine clinical practice.

## 1 Introduction

Between 2007 and 2011, 10,743 new cases of glioblastoma were diagnosed in the United Kingdom (UK) giving an annual incidence of 4.64/100,000/year [1]. Glioblastoma (GBM) is the commonest malignant brain tumor in adults, accounting for over 50% of all intrinsic brain tumors [2]. Despite improvements in surgery, radiotherapy and chemotherapy, the prognosis remains poor with the patients only having an average survival time of 14 months [3,4]. Consequently, GBM accounts for more years of life lost per patient than any other common adult cancer [5]. GBM are characterized by invasion into the surrounding brain tissue [6]. As a result, virtually all patients will progress in less than a year at the site of previous surgery and within the radiotherapy clinical target volume (CTV), [7,8].

Conventional MR imaging cannot identify the true extent of this infiltrative tumor. Several novel MR imaging techniques have been assessed for improved mapping of tumor infiltration [2] and comparative studies suggest that diffusion tensor MRI (DTI), a method sensitive to the directional diffusion of water molecules, may provide the best estimate of the invasive margin [9]. By decomposing the tensor into its isotropic component (*p*) and anisotropic component (*q*), it is possible to differentiate white matter tracts invaded by a tumor from those that have been displaced or destroyed by tumor [10]. This has been confirmed in prospective image-guided biopsy studies [11]. Our group has demonstrated that can predict sites of tumor progression [12] and can provide spatial maps of tumor infiltration zones [13] which correlate to progression free survival and location of tumor progression [14]. The ability of DTI to better identify occult tumor infiltration may improve GBM treatment planning for both surgery [15] and radiotherapy [16,17]. Uptake of the technique into routine clinical practice is hampered by the fact that segmentation of the *p* and *q* maps is time consuming and requires a degree of operator expertise.

Due to the widespread use and availability of multimodal MR imaging, segmentation of glioblastoma has been a popular area of research, often with the aim of using such segmentations as the basis of a radiomic analysis. The most successful approaches to date have utilised deep learning and in particular convolutional neural networks (CNNs), [18]. Deep neural networks (DNN) [19] have grown in popularity in the recent years due to their ability to learn complex non-linear representations of input data.

The aim of this study was to develop a tool to automate the segmentation of *p* and *q* maps, both calculated from low-resolution DTI data, together with additional contextual information from conventional MRI and perfusion MRI (or perfusion-weighted imaging (PWI)). Our research work to date confirms the clinical utility of *p* and *q* maps for the assessment of tumor infiltration. Given that image noise and limited resolution make segmentation of these maps a challenging task for a human observer, our main motivation was to assess the feasibility of automating this stage. The segmentation approach in this paper has been evaluated using DeepMedic [20], a well-established 3D CNN architecture. Using conventional MR imaging sequences, DeepMedic has been demonstrated to perform well in segmentation of the ventricles, CSF, white and grey matter [21].

## 2 Methods

### 2.1 Patients

In this study, 136 patients with supratentorial primary glioblastoma were recruited for GBM surgery from July 2010 to August 2015. Patients who had a history of previous brain tumor, cranial surgery, radiotherapy/chemotherapy, or contraindication for MRI scanning were excluded. For inclusion in the study, patients had to demonstrate a radiological diagnosis of glioblastoma on conventional MR imaging, and be suitable for surgical debulking with the intention of achieving a tumor resection of over >90%. All patients had a world health organization (WHO) performance status of 0 or 1 prior to surgery. This study was approved by the local Research Ethics Committee (10/H0308/23) and patients provided signed, informed consent. A total of 80 patients (mean age 59.4 years, range 22–76, 58 males) were studied preoperatively, yielding 80 datasets for this evaluation.

### 2.2 Dataset acquisition

Patients were imaged pre-operatively using a 3.0-T MR Magnetom system (Siemens Healthcare) with a standard 12-channel head coil. Conventional imaging included a 2D FLAIR sequence (TR/TE/TI 7840–8420/95/2500 ms; flip angle 150 ^∘^; FOV 250×200 $mm^{2}$; 25–27 slices; 1–mm slice gap; and voxel size 0.78125×0.78125×4 $mm^{3}$) and a 3D T1-weighted scan with fat suppression acquired after intravenous injection of 9 ml of gadolinium (Gadovist; Bayer Schering Pharma) (TR/TE/TI 2300/2.98/900 ms; flip angle 9^∘^; FOV 256×240 $mm^{2}$; 176–192 slices; no slice gap; and voxel size 1×1×1 $mm^{3}$). DTI data was acquired using a single-shot echo-planar sequence (TR/TE 8300/98 ms; flip angle 90^∘^; FOV 192×192 $mm^{2}$; 63 slices; no slice gap; and voxel size 2×2×2 $mm^{3}$) with multiple *b*-values (0, 350, 650, 1000, 1300, and 1600 $sec/mm^{2}$) scanned in 13 directions. PWI was acquired with a dynamic susceptibility contrast-enhancement (DSC) sequence (TR/TE 1500/30 ms; flip angle 90^∘^; FOV 192×192 $mm^{2}$; FOV 192×192 $mm^{2}$; 19 slices; slice gap 1.5 mm; voxel size of 2.0×2.0×5.0 $mm^{3}$) with 9 mL gadobutrol (Gadovist 1.0mmol/mL) followed by a 20 mL saline flush administered via a power injector at 5 mL/s. The acquisition times for the individual sequences were 4 mins and 28 secs for FLAIR, 9 mins and 26 secs for DTI, 2 mins and 21 secs for DSCI and 68 secs for the T1-weighted with contrast scan.

### 2.3 Preprocessing

DTI maps were processed with the diffusion toolbox (FDT) of FSL by applying reconstruction of diffusion tensors [22,23], normalization and eddy current correction [24,25]. Maps of fractional anisotropy (FA), mean diffusivity (MD), apparent diffusion coefficient (ADC), *p* and *q* were calculated using equations defined below [26,27].

Diffusion tensors are calculated from a symmetric 3×3 matrix as a second-order Cartesian tensor:

(1) $$D_{ij}=\left[\begin{array}{ccc} D_{xx} & D_{xy} & D_{xz}\\ D_{yx} & D_{yy} & D_{yz}\\ D_{zx} & D_{zy} & D_{zz} \end{array}\right]$$

By determining the diffusion tensors, the main eigenvalues can be calculated which indicated the fibre direction, $\lambda_{1}$, $\lambda_{2}$ and $\lambda_{3}$. By applying an eigenvalue decomposition, the resultant eigenvalues can be calculated easily and have been used in the construction of the following derivative image maps:

(2) $$FA=\sqrt{\frac{3}{2}}\frac{\sqrt{{\left(\lambda_{1}-MD\right)}^{2}+{\left(\lambda_{2}-MD\right)}^{2}+{\left(\lambda_{3}-MD\right)}^{2}}}{\sqrt{\lambda_{1}^{2}+\lambda_{2}^{2}+\lambda_{3}^{2}}}$$

(3) $$MD=\frac{1}{3}tr\left(D_{ij}\right)=\frac{\lambda_{1}+\lambda_{2}+\lambda_{3}}{3}$$

where tr represents trace of the tensor. ADC is computed in very similar calculation as the same as MD as the sum of the eigenvalues of the diffusion tensor, ADC = 3×MD [28].

is defined in a similar fashion to MD and is used to refer to the mean diffusion in a voxel, sometimes taken as the sum or average value of the tensor's diagonal elements.

(4) $$p=\sqrt{3}MD$$

(5) $$q=\sqrt{{\left(\lambda_{1}-MD\right)}^{2}+{\left(\lambda_{2}-MD\right)}^{2}+{\left(\lambda_{3}-MD\right)}^{2}}$$

*p* and MD are key representations of tensor magnitude while q and FA represent anisotropic diffusion [29]. In our previous work, p and q have been validated clinically as markers of gross tumor and invasion respectively [11]. For the DSC perfusion data, the relative cerebral blood volume (rCBV) and MR signal intensity baseline ($S_{0}$) maps were calculated using NordicICE (NordicNeuroLab, Bergen, Norway) following application of leakage correction [30]. The arterial input function was automatically defined. The baseline image in the perfusion sequence prior to contrast administration ($S_{0}$) was used for image co-registration. Defining regions of low apparent diffusion coefficient, ADC and regions of high diffusion in GBM patients generates spatially distinct tumor boundaries [31]. Therefore, in this study, rCBV and $S_{0}$ were also evaluated in combination with DTI maps to assess their effect on the predicted output segmentations [32].

**Fig. 1:**
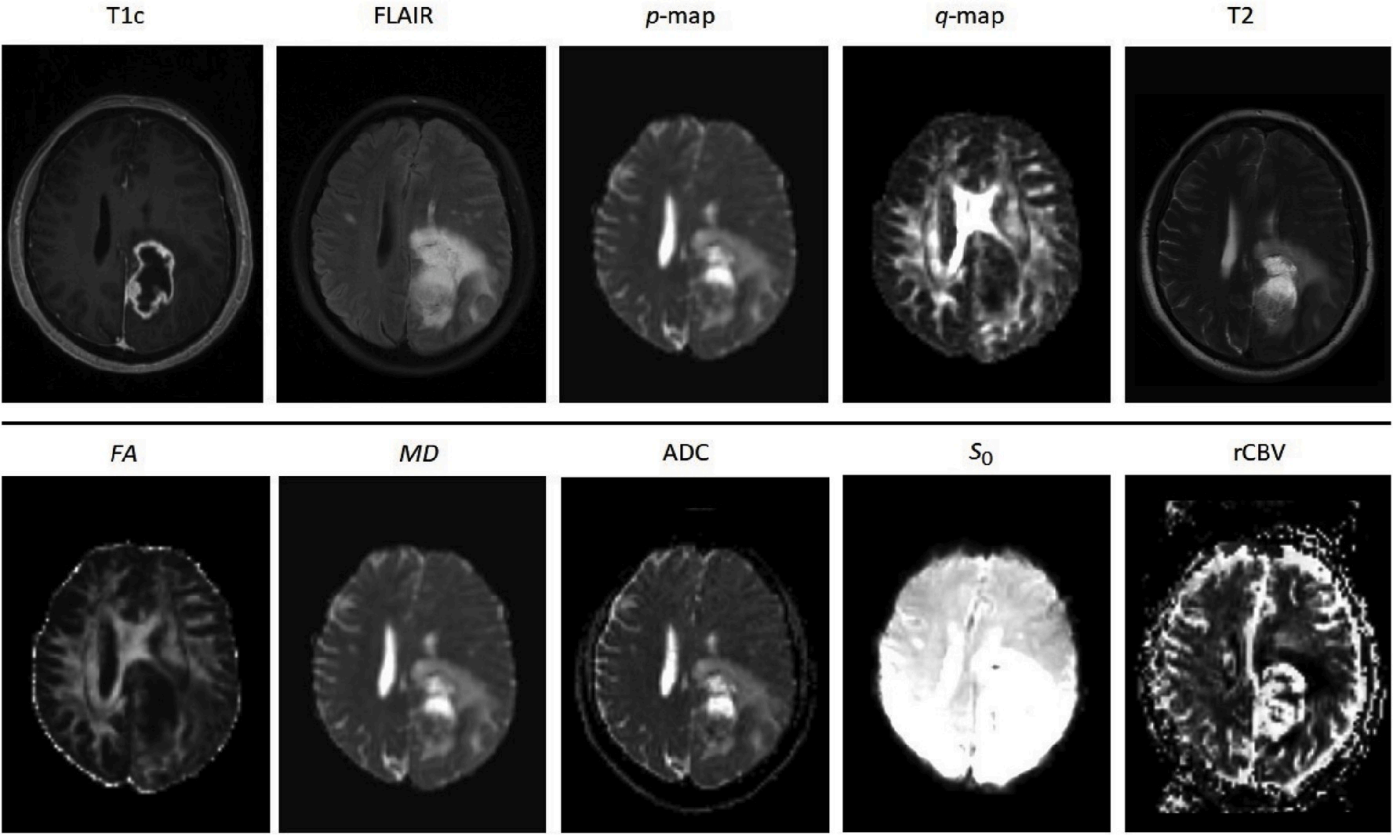
Ten different MRI modalities used in this study which consist of anatomical (T1c, FLAIR and T2), DTI (*p*, *q*, FA and MD, ADC) and PWI images ($S_{0}$ and rCBV).

Anatomical images, T1 post contrast (T1c), T2-weighted fluid attenuated inversion recovery (FLAIR), were co-registered to DTI with an affine transformation based linear image registration algorithm (FLIRT). Each dataset was resampled to a voxel size of 0.977×0.977×1 $mm^{3}$, yielding a NIFTI file with dimensions of 240×330×23 voxels. Output maps were registered to a reference axial T2 sequence using an affine transformation based rigid registration algorithm. We the ‘FLIRT’ implementation in the FSL toolbox [33]. Fig. 1 shows an example of the co-registered dataset used for one patient.

All the resulting registered images were normalized by deducting the mean value from each pixel and dividing by the standard deviation of intensity values.

### 2.4 Ground truth

The ground truth segmentations for this dataset were manually delineated on four modalities: enhancing tumor (T1c, FLAIR, *p* and *q*), non-enhancing tumor (FLAIR), abnormal isotropic (*p*) and anisotropic diffusion (*q*). The manual regions of interest (ROIs) were independently contoured by three observers: a neurosurgeon with > 8 years of experience (CL), a neurosurgeon with > 9 years of experience (JLY), and a researcher with > 4 years of brain tumor image analysis experience (NRB). Segmentations and masks were generated using 3D Slicer (v4.6.2) [34]. The observers performing manual segmentation were blinded to the model construction and validation phases.

**Fig. 2:**
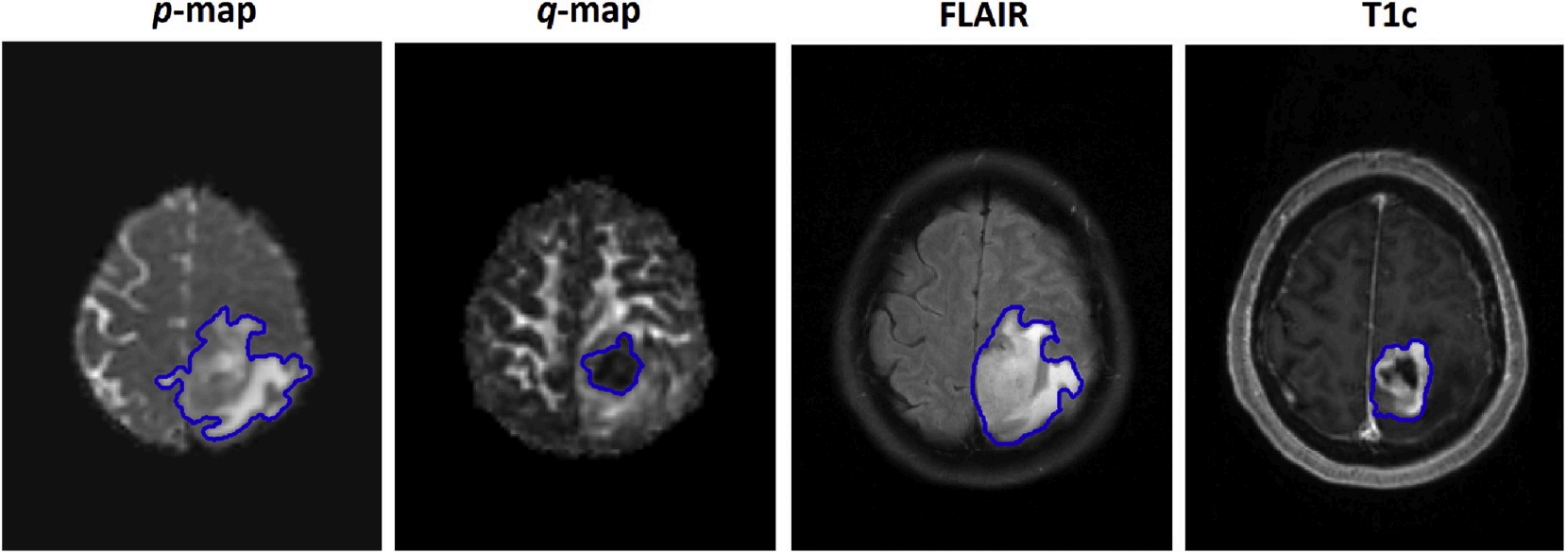
Four different MRI modalities and their relevant ground truth segmentations from the same patient. The images demonstrate distinct tumor compartments visualised by each MRI sequence. The *q*-map has been shown previously to show areas of high tumor cell density and the *p*-map shows invasive regions. The T1c and FLAIR regions demonstrate the enhancing, necrotic, and non-enhancing tumor components respectively.

Majority voting was used to develop consensus of the ground truth where there was significant disagreement. Previous studies have shown excellent agreement using this method [35]. Fig. 2 demonstrates four different contours delineated for the same slice position on four image sequences.

### 2.5 Segmentation methodology

**Fig. 3:**
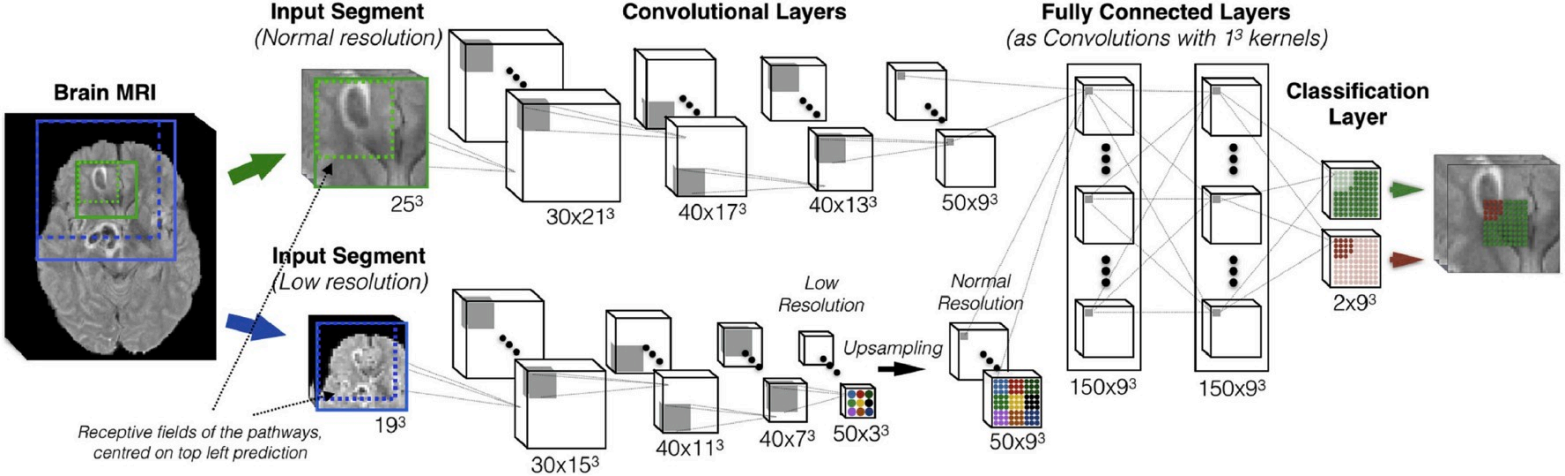
The DeepMedic convolutional neural network architecture includes a multi-scale 3D CNN with two convolutional pathways of 11-layers. Feature extraction layers consist of size $5^{3}$ kernels (Adapted from Fig. 5 in Ref. [21].

For automatic segmentation, we used DeepMedic, an 11-layer multi-scaled 3D CNN architecture that has been used for medical image segmentation and demonstrated to be robust in similar applications [20,21]. The architecture of DeepMedic is shown in Fig. 3. Briefly, it consists of two parallel convolutional pathways, four feature extraction layers with $5^{3}$ kernels for feature extraction, two fully connected layers and a final classification layer. The dual pathway architecture allows for multi-scale processing of the input images to achieve a large receptive field for the final classification, while keeping the computational cost low. The first pathway operates on the original image, and the second one operates on a down-sampled version.

**Fig. 4:**
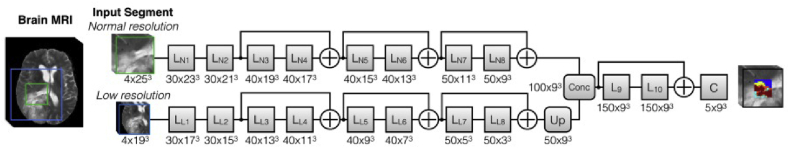
The DeepMedic architecture extended with residual connections. In this architecture residual connections are added between the outputs of every two layers, except for the first two layers of each pathway to direct the network away from raw intensity values (Adapted from Fig1 in Ref. [20].

DeepMedic was extended with residual connections [36] to improve performance. These additional connections facilitate preservation of the flowing signal, thus enabling training of very deep neural networks, (summarized in Fig. 4), [20].

**Fig. 5:**
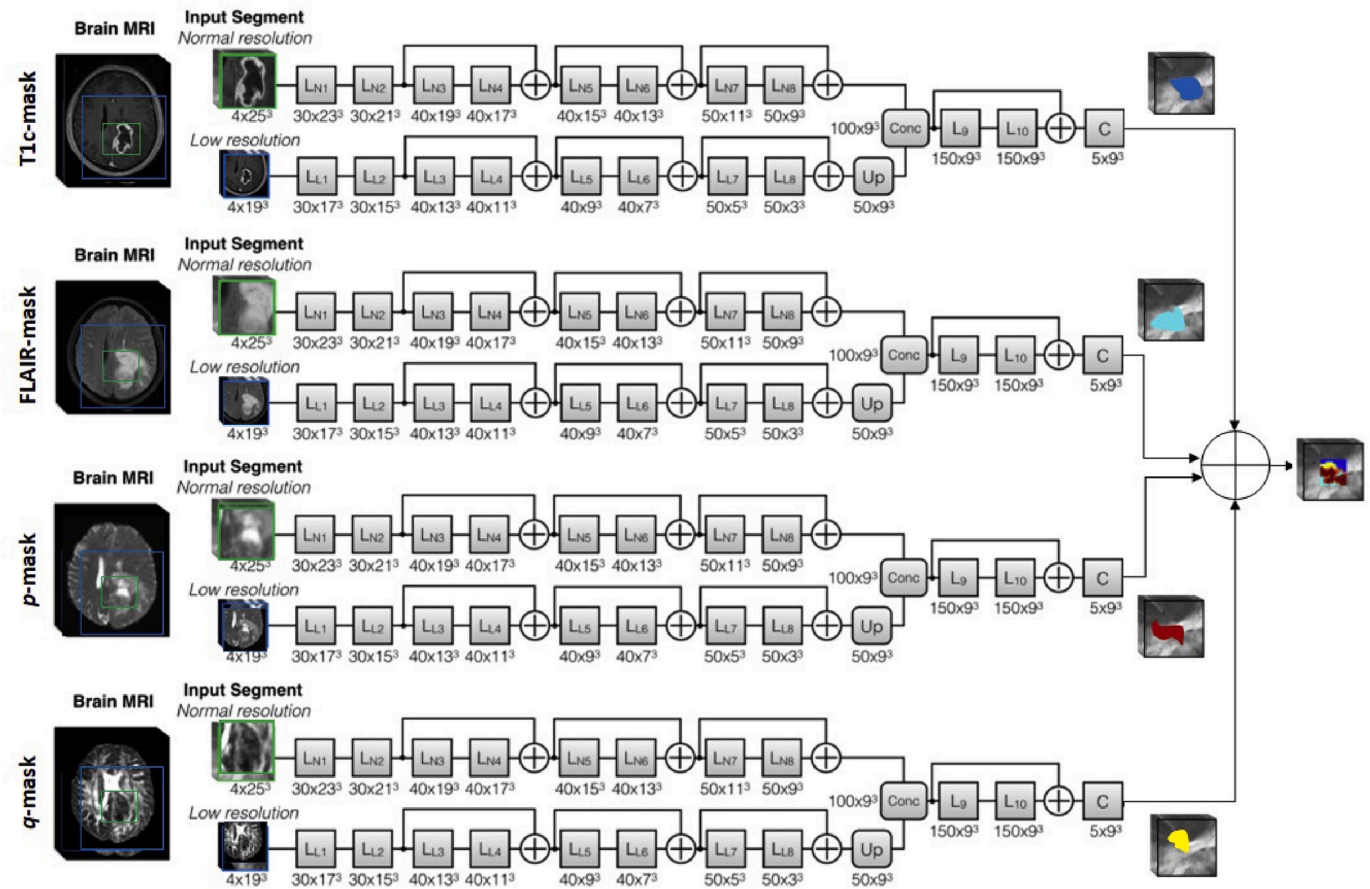
The framework of our architecture extension to DeepMedic [20], each ground truth is considered separately from other ground truths. Depending on the number of inputs to the network, this number of pathways can be adjusted (we have shown this extension to four inputs here for illustration).

Data augmentation was performed via reflection with respect to the mid-sagittal plane.

#### 2.5.1 Extension of DeepMedic

In this study, each ground truth is considered individually with its own image channel and not merged as in most implementations of the network. This is because each labelled region is evaluated in the absence of other regions. The study dataset included 10 distinct image sequences derived from diffusion and perfusion imaging as well as standard anatomical sequences. We therefore modified the input layers of DeepMedic, to utilise a multi-pathway architecture, assessing different combinations of image sequences in turn. The motivation for this approach was to determine the optimum set of input channels to provide segmentation of *p* and *q* abnormalities. Fig. 5 demonstrates the framework of our extension to DeepMedic. Regardless of the number of paths utilised, the final segmentations were merged to show the multi-class segmentation results on a single image.

#### 2.5.2 Experiment setup

Table 1 shows the chosen combination of segmentation models used at training time. Training was performed using an implementation of Deepmedic on Tensorflow, using an NVIDIA Titan Xp graphics card [37]. Training time for each model varied from 4 to 8 h.

**Table 1:** Different models in the experiment setup for evaluating the multi-scale segmentation of GBM.

Model	MR-Sequence(s)	Ground-Truth
1	*p-q*-FLAIR-T1c-T2-*FA-MD*-ADC-$S_{0}$-rCBV	*p*-mask
2	*p-q*-FLAIR-T1c-T2-*FA-MD*-ADC-$S_{0}$-rCBV	*q*-mask
3	*p-q*-FLAIR-T1c-T2-*FA-MD*-ADC-$S_{0}$-rCBV	FLAIR-mask
4	*p-q*-FLAIR-T1c-T2-*FA-MD*-ADC-$S_{0}$-rCBV	T1c-mask
5	*p-q*-FLAIR-T1c	*p*-mask
6	*p-q*-FLAIR-T1c	*q*-mask
7	*p-q*-FLAIR-T1c	FLAIR-mask
8	*p-q*-FLAIR-T1c	T1c-mask
9	*p-q*	*p*-mask
10	*p-q*	*q*-mask
11	FLAIR-T1c	FLAIR-mask
12	FLAIR-T1c	T1c-mask
13	*p*	*p*-mask
14	*q*	*q*-mask
15	FLAIR	FLAIR-mask
16	T1c	T1c-mask

Segmentation performance was evaluated on the same combinations shown in Table 1. For each individual model, a single ground truth was chosen to train the network, and as the output prediction. Finally, the segmentation outcome of all models with the same training sequences were merged to visualize different tumor compartments. For instance, Models 1–4 use ten different sequences as their inputs (8 different data types), which contain all the anatomical, DTI and PWI images in the dataset. It is self-evident from their definitions that MD, ADC and *p* sequences all represent the isotropic component of the diffusion tensor, but with different output scaling [28]. We elected to include them as separate input sequences. This had the effect of increasing the training data size at the cost of potential biased, as there is a threefold weighting towards the mean diffusion signal. The motivation for this ‘hold-out’ technique was to assess the incremental benefit of different forms of MR image sequence on segmentation performance. Models 5–8 use only four related sequences to the four ROIs in the absence of other DTIs or PWI images. Models 9–12 pairs the DTI and anatomical ones to evaluate their relevant ROIs, and Models 13–16 evaluates them as individual image sequences in the absence of any other image data. The evaluation of the obtained segmentations is demonstrated in qualitative and quantitative form. The qualitative analysis has been performed by expert. The dataset for all modules were divided into 40 patients for training, 10 for validation and 30 patients for testing. In each model the number of images varies due to the number of modalities involved in the analysis.

#### 2.5.3 Evaluation of segmentation

The segmentation results were evaluated using Dice coefficient (DC), [38].

## 3 Results

### 3.1 Quantitative analysis

The overall results output by each model are shown in Table 2. In this table, the number of patients used in training, validation and test sets has been listed. The difference in the number of images available for training and testing in each experiment affects the DC for the testing sets. The average DC for the training and test sets in each model are shown highlighting poor DC performance for the smaller datasets.

**Table 2:** Dice coefficient performance of modified DeepMedic for different models listed in Table 1.

Model	# Training set	# Validation set	# Test set	Average train DC	Average test DC (± SD)
1	8280 Slices (40 patients)	2070 Slices (10 patients)	6210 Slices (30 patients)	0.67	0.71 (± 0.13)
2	8280 Slices (40 patients)	2070 Slices (10 patients)	6210 Slices (30 patients)	0.68	0.66 (± 0.21)
3	8280 Slices (40 patients)	2070 Slices (10 patients)	6210 Slices (30 patients)	0.73	0.78 (± 0.11)
4	8280 Slices (40 patients)	2070 Slices (10 patients)	6210 Slices (30 patients)	0.82	0.82 (± 0.17)
5	3680 Slices (40 patients)	920 Slices (10 patients)	2760 Slices (30 patients)	0.63	0.69 (± 0.11)
6	3680 Slices (40 patients)	920 Slices (10 patients)	2760 Slices (30 patients)	0.65	0.65 (± 0.21)
7	3680 Slices (40 patients)	920 Slices (10 patients)	2760 Slices (30 patients)	0.77	0.77 (± 0.15)
8	3680 Slices (40 patients)	920 Slices (10 patients)	2760 Slices (30 patients)	0.83	0.81 (± 0.17)
9	1840 Slices (40 patients)	460 Slices (10 patients)	1380 Slices (30 patients)	0.51	0.49 (± 0.25)
10	1840 Slices (40 patients)	460 Slices (10 patients)	1380 Slices (30 patients)	0.37	0.38 (± 0.27)
11	1840 Slices (40 patients)	460 Slices (10 patients)	1380 Slices (30 patients)	0.80	0.75 (± 0.16)
12	1840 Slices (40 patients)	460 Slices (10 patients)	1380 Slices (30 patients)	0.80	0.76 (± 0.23)
13	920 Slices (40 patients)	230 Slices (10 patients)	690 Slices (30 patients)	0.42	0.37 (± 0.23)
14	920 Slices (40 patients)	230 Slices (10 patients)	690 Slices (30 patients)	0.46	0.36 (± 0.28)
15	920 Slices (40 patients)	230 Slices (10 patients)	690 Slices (30 patients)	0.69	0.67 (± 0.19)
16	920 Slices (40 patients)	230 Slices (10 patients)	690 Slices (30 patients)	0.58	0.56 (± 0.24)

Table 2 shows the ratio of training, validation and testing sets for each model in Table 1 as well as their relevant DC. In all models the same patients were chosen to set up the experiments while the number of input images were different due to using different modalities per patient. For instance, Model 1 consists of 10 training channels which are equivalent to 8280 Slices of 40 patients from 10 different MRI modalities (10×23×40). The DeepMedic architecture used in this work, incorporates a data shuffle at the start of each epoch to avoid overfitting, which can be seen from the close behavior of DC for training and test sets in Table 2.

**Fig. 6:**
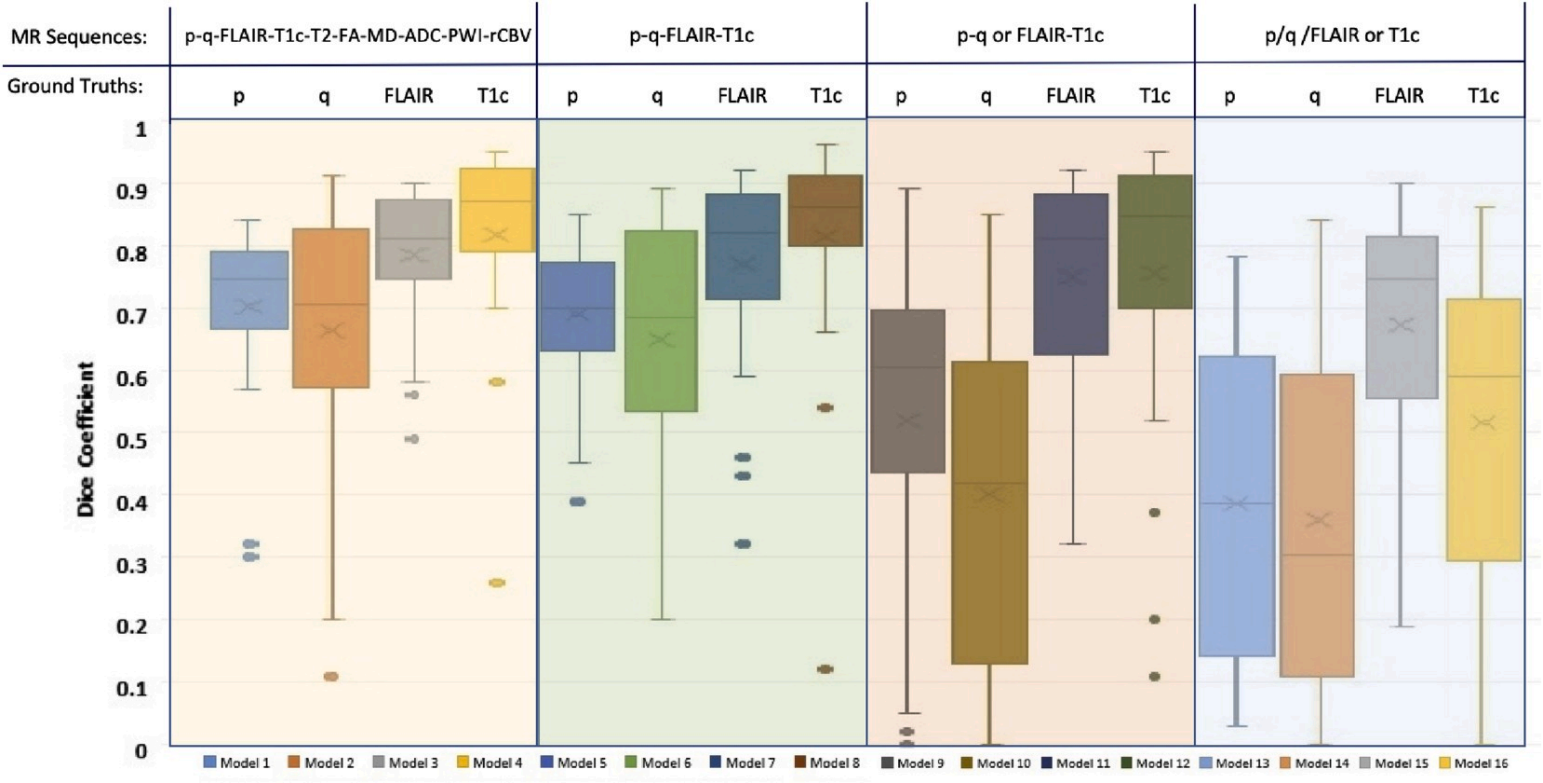
Box plots of the similarity scores (DC) between the image segmentation output by all models and the reference ground truth for each ROI. Different colored boxes refer to the number of inputs in the extended DeepMedic shown in Fig. 5.

Fig. 6 illustrates the DC values for the output segmentation results for the test sets for each combination in Table 1. The results demonstrate good performance of the DeepMedic architecture on the available dataset. Encouragingly, the performance for the DTI segmentation improves greatly when it is combined with the conventional MR images such as FLAIR and T1c. It is interesting to observe the performance of Models 1–4, as they utilise additional input information from 10 channels (8 data types) to train the network, though it should be borne in mind there may be a bias towards diffusion signal information in these models.

Models 5–8, which make use of contrast enhanced T1, FLAIR and two tensor decomposition sequences, appeared to demonstrate the best segmentation performance among all evaluated models, suggesting that the *p* and *q* maps encapsulate most of the image information that is added by diffusion imaging. Models 13–16 have lower performance due to limited availability of training data. The results of models 9, 10, 13 and 14 demonstrate poor segmentation performance for *p* and *q* maps. This illustrates that spatial context from other image sequences is needed to segmentation of DTI maps.

Across all model runs, we observe an increase in DC as the number of input channels is increased. Non-parametric Wilcoxon sign rank testing was performed by pairwise comparison of Models 5–8 with Models 9–12 and Models 13–16 [39]. The test shows a significant difference (p< 0.01) in model performance as measured by DC values.

Also, a general point should be made about the performance drop observed when training the network with DTI *p* and *q* maps only (Models 9–10 and Models 13–14) in comparison to training them along anatomical data. This can be improved as part of future work by adding more data augmentation methods using generative models.

### 3.2 Qualitative analysis

**Fig. 7:**
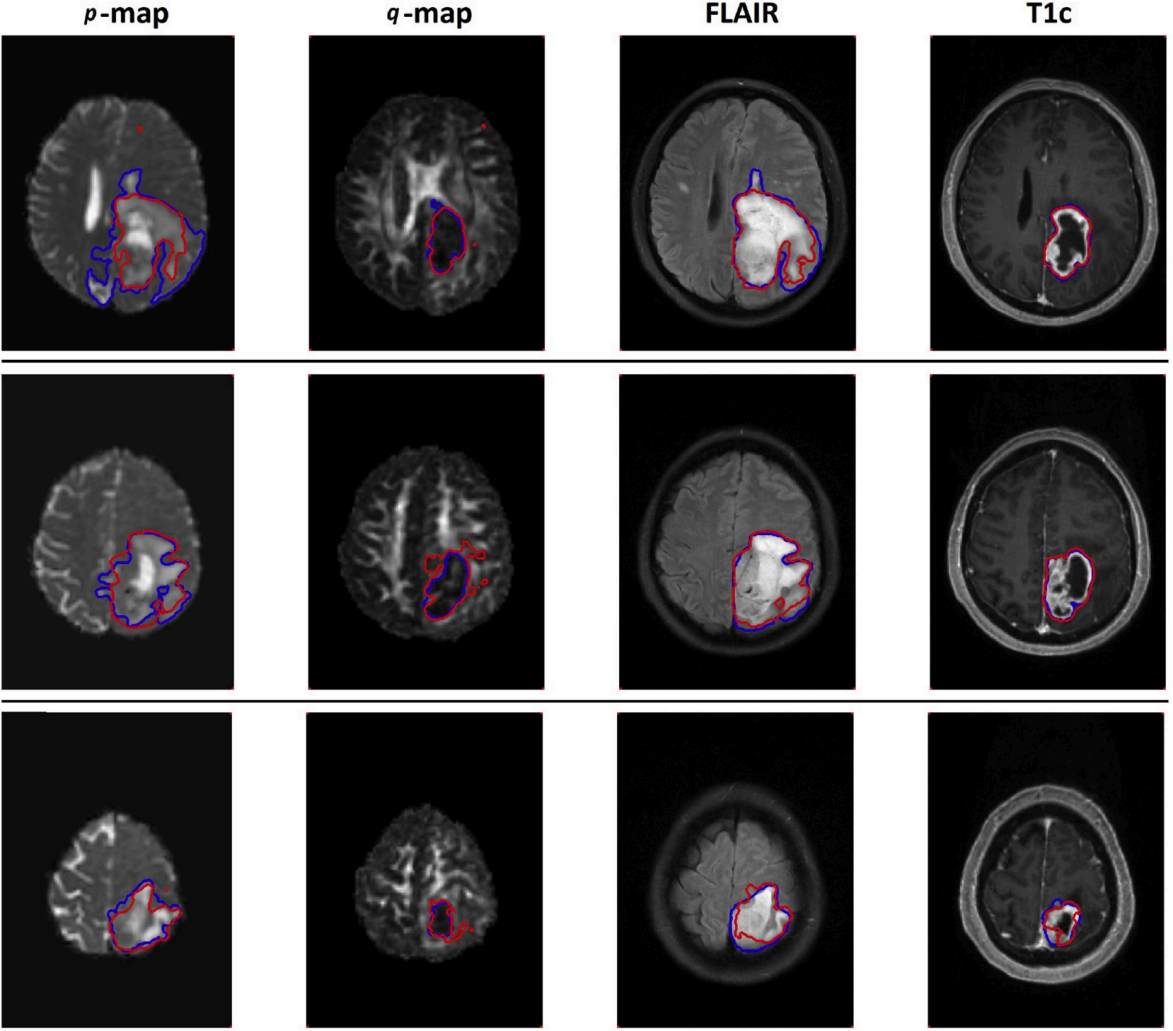
Three example slices of the same patient, with associated ground truth and automatic segmentations. Blue shows the ground truth delineated by the expert clinician and the red contours represent the outcome of our segmentations. (For interpretation of the references to colour in this figure legend, the reader is referred to the Web version of this article.)

Fig. 7 shows three representative slices from the same patient, with associated ground truth and automatic segmentations. Qualitative analysis of the output segmentation results confirms that segmentation performance is enhanced by combining information from DTI *p* and *q* maps with conventional FLAIR and T1c. We found that the architecture is capable of precise segmentation of both small and large lesions on each image modality.

**Fig. 8:**
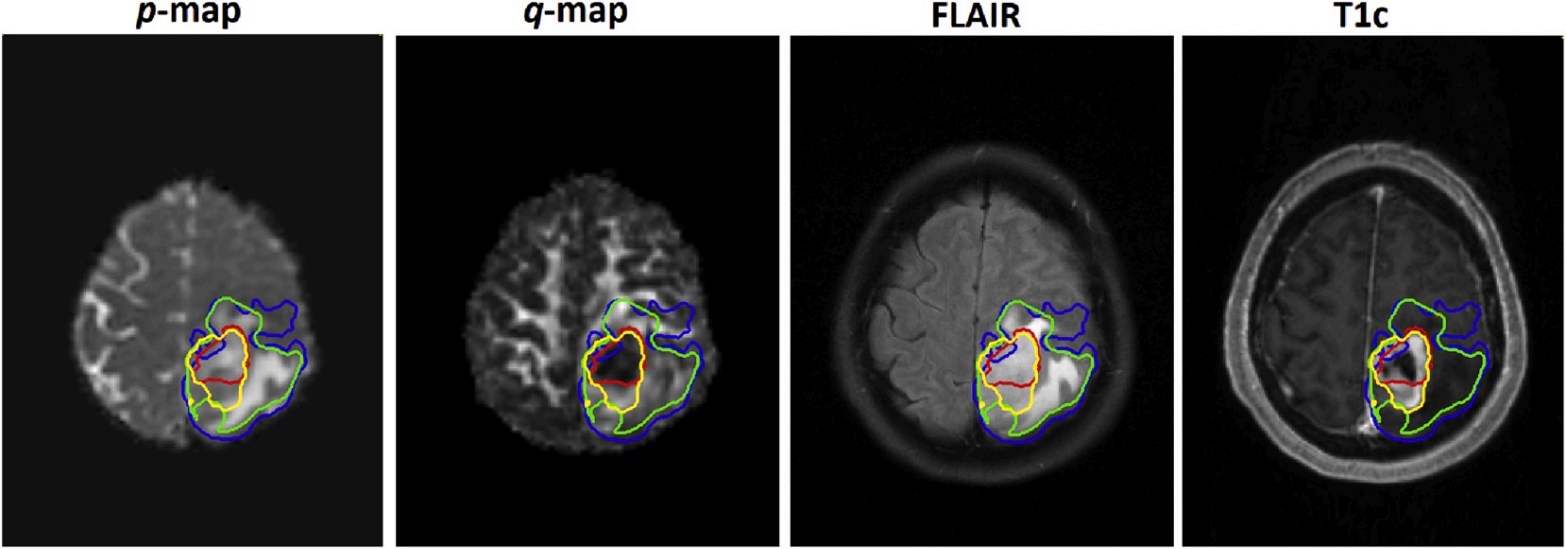
Demonstrating all four segmentations obtained from different models (Models 5–8), where the segmentation in blue is for *p*, red (DC = 80%) for *q*, green (DC = 85%) for FLAIR (DC = 89%) and yellow for T1c (DC = 80%). (For interpretation of the references to colour in this figure legend, the reader is referred to the Web version of this article.)

Fig. 8 illustrates all four segmentations generated by models 5–8 on top of each image sequence. The contours are colour coded in blue for *p* map, red for *q* map, green for FLAIR and yellow for T1c. Provision all of these segmentations automatically could assist clinicians in appreciating the different tumor compartments observed in a typical GBM.

These initial results suggest that the discriminative power of the learned features to segment *p* and *q* DTI-maps could match human expert observer performance. However, this proof of concept has been implemented on a highly complex framework with a large computational burden. As part of future work, we will attempt to limit the number of trainable parameters by sharing weights across layers. We will also assess other deep learning frameworks for large scale data such as the U-net implementation of [40] and assess the role of supervised machine learning models for smaller datasets as implemented in Ref. [[41], [42], [43]].

## 4 Discussion

This proof-of-concept study shows that automatic segmentation of subcomponents of GBM can be performed through a novel application of an existing CNN architecture that has been optimised for medical image segmentation. Furthermore, we have established that integrating DTI based *p* and *q* with conventional MR image sequences produces results with potential clinical utility. Since our goal was to optimize segmentation of *p* and *q* maps from different combinations of image sequences, we did not individualize the architecture for each image sequence, employing instead the default architecture of DeepMedic [44].

Signal changes seen with conventional anatomical MRI (T1-weighted and T2-weighted/FLAIR images) are not specific to the pathological changes seen with tumors. They lack sensitivity to the occult invasive growth of gliomas. DTI provides more a sensitive and specific biomarker for the disruption of white matter tracts caused by tumor invasion. Our previous image-guided biopsy study has shown that DTI *p* and *q* maps achieved a sensitivity of 98% and specificity of 81% in differentiating gross tumor and tumor infiltration [11,45]. Furthermore, DTI *p* and *q* maps were subsequently used to predict tumor recurrence patterns [27] and have been correlated with IDH-1 mutation status, a driver mutation of gliomas [35]. A higher extent of resection of the DTI *p* and *q* abnormalities has also been shown to correlate with better patient prognosis [15,46]. This supports the importance of integrating DTI derived parametric maps into clinical decision-making process. With this simple, multi-sequence framework constructed in DeepMedic, the results obtained provides proof of concept that automatic segmentation of *p* and *q* abnormalities could speed up the image processing workflow and has the potential to assist clinicians with interpretation of DTI data. Current clinical management of GBM relies heavily on MRI images. Yet more advanced MR (DTI and PWI) are rarely used for routine management. The difficulty in automating their segmentation have prevented use in routine clinical care. The limited studies in this field have included DTI alone or in combination with few other imaging modalities. In this study we provide initial evidence that these low-resolution sequences can be segmented automatically when combined with other imaging modalities. This will allow automatic GBM segmentation of the DTI to allow interventional studies that change surgical and radiotherapy planning volumes.

## 5 Conclusions

We have demonstrated that a multi-channel architecture provides the best segmentation of DTI based *p* and *q* maps. The network used in this proof of concept study has been trained and tested on a small clinical dataset. Validation of the network on an independent dataset would be required to confirm the utility and generalisability of this approach.
